# Association of a Sweetened Beverage Tax With Purchases of Beverages and High-Sugar Foods at Independent Stores in Philadelphia

**DOI:** 10.1001/jamanetworkopen.2021.13527

**Published:** 2021-06-15

**Authors:** Sara N. Bleich, Caroline G. Dunn, Mark J. Soto, Jiali Yan, Laura A. Gibson, Hannah G. Lawman, Nandita Mitra, Caitlin M. Lowery, Ana Peterhans, Sophia V. Hua, Christina A. Roberto

**Affiliations:** 1 Department of Health Policy and Management, Harvard T.H. Chan School of Public Health, Boston, Massachusetts; 2 Department of Medical Ethics and Health Policy, University of Pennsylvania Perelman School of Medicine, Philadelphia; 3 Division of Chronic Disease Prevention, Philadelphia Department of Public Health, Philadelphia, Pennsylvania; 4 Department of Biostatistics, Epidemiology and Informatics, University of Pennsylvania Perelman School of Medicine, Philadelphia; 5 Department of Health Policy and Management, Johns Hopkins Bloomberg School of Public Health, Baltimore, Maryland

## Abstract

**Importance:**

The relationship between a sweetened beverage tax and changes in the prices and purchases of beverages and high-sugar food is understudied in the long term and in small independent food retail stores where sugar-sweetened beverages are among the most commonly purchased items.

**Objective:**

To examine whether a 1.5 cent-per-fluid-ounce excise tax on sugar- and artificially sweetened beverages Philadelphia, Pennsylvania, was associated with sustained changes in beverage prices and purchases, as well as calories purchased from beverages and high-sugar foods, over 2 years at small independent stores.

**Design, Setting, and Participants:**

This cross-sectional study used a difference-in-differences approach to compare changes in beverage prices and purchases of beverages and high-sugar foods (candy, sweet snacks) at independent stores in Philadelphia and Baltimore, Maryland (a nontaxed control) before and 2 years after tax implementation, which occurred on January 1, 2017. Price comparisons were also made to independent stores in Philadelphia’s neighboring counties.

**Main Outcomes and Measures:**

Changes in mean price (measured in cents per fluid ounce) of taxed and nontaxed beverages, mean fluid ounces purchased of taxed and nontaxed beverages, and mean total calories purchased from beverages and high-sugar foods.

**Results:**

Compared with Baltimore independent stores, taxed beverage prices in Philadelphia increased 2.06 cents per fluid ounce (95% CI, 1.75 to 2.38 cents per fluid ounce; *P* < .001), with 137% of the tax passed through to prices 2 years after tax implementation, while nontaxed beverage prices had no statistically significant change. A total of 116 independent stores and 4738 customer purchases (1950 [41.2%] women; 4351 [91.8%] age 18 years or older; 1006 [21.2%] White customers, 3185 [67.2%] Black customers) at independent stores were assessed for price and purchase comparisons. Purchases of taxed beverages declined by 6.1 fl oz (95% CI, −9.9 to −2.4 fl oz; *P* < .001), corresponding to a 42% decline in Philadelphia compared with Baltimore; there were no significant changes in purchases of nontaxed beverages. Although there was no significant moderation by neighborhood income or customer education level, exploratory stratified analyses revealed that declines in taxed beverage purchases were larger among customers shopping in low-income neighborhoods (−7.1 fl oz; 95% CI, −13.0 to −1.1 fl oz; *P* = .001) and individuals with lower education levels (−6.9 fl oz; 95% CI, −12.5 to −1.3 fl oz; *P* = .001).

**Conclusions and Relevance:**

This cross-sectional study found that a tax on sweetened beverages was associated with increases in price and decreases in purchasing. Beverage excise taxes may be an effective policy to sustainably decrease purchases of sweetened drinks and calories from sugar in independent stores, with large reductions in lower-income areas and among customers with lower levels of education.

## Key Points

**Question:**

How has the 2017 Philadelphia beverage tax factored into longer-term changes in beverage prices and purchases in independent food retail stores based on observational data?

**Findings:**

This cross-sectional study found that, 2 years after tax implementation, price audits of stores showed 137% of the tax was passed through to prices and bag checks indicated a 42% decline in volume of taxed beverages purchased in Philadelphia compared with Baltimore. Total calories purchased from beverages and high-sugar foods declined, suggesting food substitution did not offset beverage declines.

**Meaning:**

These findings suggest a city-level beverage excise tax was associated with persistent declines in purchases of sweetened drinks and calories from sugar in independent stores.

## Teaser

This cross-sectional study assesses the association of a sweetened beverage tax imposed by the city of Philadelphia, Pennsylvania, with price changes on beverages in small independent retail stores and examines changes in purchases of sweetened beverages and high-sugar foods after implementation of the tax.

## Introduction

Beverage taxes are a promising policy to reduce sugar-sweetened beverage (SSB) consumption. SSBs are more commonly consumed by communities of color and low-income populations,^1,2,3^ and excess consumption is associated with poor health outcomes.^4,5,6,7^ As of 2020, beverage taxes—ranging from 1 to 2 cents per fluid ounce—were implemented in 7 US localities. A tax in Philadelphia, Pennsylvania, enacted January 1, 2017, was for 1.5 cents per fluid ounce.

Mounting research suggests that beverage taxes are consistently associated with increased prices,^8,9,10,11,12,13,14,15,16,17,18,19,20^ and reductions in the volume of taxed beverages sold^13,14,15,19,20,21,22,23,24^ with considerable variation by retailer type and tax jurisdiction. There is some evidence that beverage taxes are associated with reductions in self-reported consumptions of SSBs, although results are mixed and many studies are limited by small sample sizes.^22,24,25,26,27^ One year after Philadelphia’s 1.5-cent-per-fluid-ounce tax on sugar- and artificially sweetened beverages, we found that small, independent stores passed-through 120% of the tax to prices and the fluid ounces of taxed beverages per purchase declined by 39%.^14^ Tax effects at small, independent stores have been understudied, despite SSBs being among the most commonly purchased items at these stores^28,29,30^ and the many shopping trips made to these stores in urban and low-income areas.^28^ Small business owners are also key stakeholders in beverage tax policy discussions, underscoring the importance of understanding small store sales in response to a tax. There are also limited data on whether initial reductions in sweetened beverage sales in response to these taxes persist beyond the first year. Mexico’s countrywide tax was associated with persistent reductions in taxed beverage sales 2 years later,^23^ but US city tax effects might lessen over time given the ability to cross city lines to avoid the tax. In addition, although 1 published study has found no evidence of substitution to high-calorie foods or alcohol in place of SSBs 1 year after Philadelphia’s tax,^31^ no studies have examined potential substitution over the longer term (ie, ≥2 years).

This study fills these gaps by evaluating the association between implementation of the Philadelphia tax and changes in beverage prices and fluid ounces purchased, as well as total calories purchased from beverages and high-sugar foods, 2 years posttax in a large sample of small, independent stores. We hypothesized the tax would be associated with significant beverage price increases and reductions in taxed beverage sales, with limited substitution to high-sugar foods. Our secondary aims examined differences by beverage sweetener status and container size as well as neighborhood income in neighborhoods where stores are located and customer education level.

## Methods

### Study Design

In this cross-sectional study, we used a difference-in-differences approach to examine pretax vs posttax beverage prices, fluid ounces purchased, and total calories purchased from beverages and high-sugar foods at small independent food retailers in Philadelphia compared with Baltimore, Maryland. Baltimore was the control city because it is geographically close to Philadelphia but does not border it and has a similar demographic composition.^32^ In prior work, we observed parallel trends in beverage volume sales among large, chain retailers in 2016 and therefore assumed it was similar for independent stores.^15^

Stores were included if they sold at least 3 of 31 beverages assessed on store audit forms (methods and forms published elsewhere^14^). Trained research assistants collected data on beverage prices and customer purchases at baseline (October-December 2016) right before the January 1, 2017, tax implementation date; 6 months after tax implementation (June-August 2017); 12 months after implementation (October-December 2017); and 24 months after implementation (October-December 2018). This article presents results from data sampled 24 months following tax implementation. Price data were also collected at small independent stores in untaxed, neighboring Pennsylvania counties to determine whether the tax was associated with prices at bordering stores.

Details on beverage categorization, data collection, and measures are described elsewhere^14^ and summarized below. This research was approved by the institutional review boards of the University of Pennsylvania, Harvard T.H. Chan School of Public Health, and the Philadelphia Department of Public Health. A waiver of informed consent was approved for adult participants. For adolescent participants, a waiver of parental consent was approved and verbal assent was obtained. This study followed the Strengthening the Reporting of Observational Studies in Epidemiology (STROBE) reporting guideline.

### Beverage and Food Categorization

Beverages from customer bag checks and inspection of receipts were categorized by tax status (taxed vs nontaxed) based on Philadelphia’s beverage tax regulations (eAppendix 1 in the Supplement), sweetener status (sugar-sweetened, artificially sweetened, or unsweetened), and container size (individual- or family-sized). Foods were sorted into 15 categories. We identified 2 high-sugar food categories (candy and sweet snacks [eg, cookies]) as potential ready-to-eat sweet substitutes for SSBs. We conducted online searches to identify the calorie content, grams of sugar per serving, and serving size (fluid ounces for beverages, grams for food) for all beverages and high-sugar foods that were likely sweet substitutes, prioritizing information provided on brand websites (eAppendix 1 in the Supplement).

### Beverage Prices: Procedures and Measures

Study stores were identified through random sampling from ReferenceUSA.^33^ Because many stores on the ReferenceUSA list were permanently closed, we supplemented this approach by identifying replacement independent stores in close proximity to the closed randomly sampled ones. We defined stores in census tracts with 30% or more of the population living in poverty as “low-income” and the rest as “other income.” When prices were unlisted, staff asked store employees or purchased the beverage. eAppendix 1 in the Supplement and our previously published study^14^ have further details.

### Customer Purchases: Procedures and Measures

We assessed customer purchases at 58 small independent retailers in Philadelphia and 63 in Baltimore (this includes 78 stores where we also obtained price data). Trained research assistants stood outside stores on weekdays at 3 regular times a day for approximately 2 months at each time-point and asked every customer leaving stores who appeared at least 13 years old if they purchased any food or beverage item and would allow a bag check. A gift valued at $3 or less was offered. Purchase data missing beverage tax status, price, quantity, or number of ounces were excluded (98 customers). Beverage concentrates were also excluded because of small numbers purchased (8 customers in Philadelphia, 10 in Baltimore).

Research assistants recorded detailed descriptions of each food and beverage item purchased including volume, quantity, and price and asked customers to self-report the total amount spent, demographic characteristics, and their frequency of visiting the store, SSB consumption (assessed by 1 survey question), and SSB purchasing in neighboring counties. Institutional review board permission to waive consent required only allowing 3 racial categories for self-identification (ie, Black, White, Other) and 1 ethnicity (Hispanic/Non-Hispanic). These data were collected to allow us to compare the demographic composition of our samples over time and between our intervention and control sites.

### Outcome Variables

For price analyses, the primary outcome was the change in mean beverage price, in cents per fluid ounce, of audited taxed and nontaxed beverages between baseline and 24 months posttax. Energy drinks were excluded from price analyses due to their much higher mean price per fluid ounce (eAppendix 1 in the Supplement). For customer purchase analyses, the primary outcomes were the change in purchased fluid ounces of taxed and nontaxed beverages per person (ie, the sum of all taxed or nontaxed beverages purchased per customer purchase assessment) and change in total calories from beverages and high-sugar food purchases (calculated using the recorded size per item and nutrient information; to convert fluid ounces to mL, multiply by 30). Secondary outcomes were the change in beverage price and total purchased beverage ounces by sweetener type (for taxed beverages only), container size, store income level, and customer education level. Lower education was defined as having a high school degree, General Educational Development diploma, or less, while higher education included some college or more. We also examined changes in grams of sugar purchased and total amount spent (including food/beverages and other items).

### Statistical Analysis

For each study outcome, we used a difference-in-differences approach, using generalized linear mixed effects modeled with a normal distribution and identity link. Robust standard errors and random intercepts for stores were used to estimate changes in mean beverage price and purchase volume and calories purchased. Each model included a binary indicator for the posttax vs pretax period, location, and their interaction (the difference-in-differences estimate of the tax effect size). The main analyses compared Philadelphia with Baltimore. We also compared change in beverage prices between the counties bordering Philadelphia and Baltimore. To test the balance in sample composition during the study period, models were adjusted for characteristics identified a priori to influence purchases at small, independent stores including gender, race, ethnicity, education, who the purchase was for, frequency visiting the store, city residency, and total reported spending. Unadjusted results are presented throughout because adjusted results were not meaningfully different (eAppendix 3 in the Supplement). All analyses were 2-sided tests using an α = .05, and beverage price and volume *P* values were adjusted using Bonferroni corrections to account for 2 comparisons of the tax by beverage types and 4 comparisons of the tax by customer demographic characteristics and store location. Sugar and calorie *P *values were Bonferroni corrected to account for 2 comparisons of purchases by customer characteristics and store location demographics. We examined whether income and education moderated tax effect sizes with interactions by indicators for posttax vs pretax period and location (the triple difference-in-difference estimate of the tax effect size between the 2 groups). Because differences by income and education are of great public health interest, we also conducted exploratory stratified analyses with these variables. Sensitivity and subgroup analyses appear in the eAppendices 2, 3, and 4 in the Supplement. Analyses were conducted using Stata 15.1 (StataCorp) and replicated by a second analyst (J.Y.).

## Results

### Change in Beverage Price

A total of 116 independent stores and 4738 customer purchases (1950 [41.2%] women; 4351 [91.8%] age 18 years or older; 1006 [21.2%] White customers, 3185 [67.2%] Black customers) at independent stores were assessed for price and purchase comparisons. The characteristics of the price audited stores were similar across Philadelphia and Baltimore (eAppendix 1 in the Supplement). There was a 2.06 cents per fl oz (95% CI, 1.75 to 2.38 cents per fl oz; *P* < .001) increase for taxed beverages in Philadelphia compared with Baltimore, an increase of 33.3%, indicating a 137.3% pass-through of the tax (Table 1). There were no statistically significant changes in the prices of nontaxed beverages. The price increase for SSBs was 2.03 cents per fluid ounce (95% CI, 1.68 to 2.37 cents per fl oz; *P* < .001), or 32.7%, and for artificially sweetened beverages was 2.22 cents per fluid ounce (95% CI, 1.35 to 3.08 cents per fl oz; *P* < .001), for a 36.1% increase. For taxed individual size drinks, the price increase was 1.98 cents per fluid ounce (95% CI, 1.69 to 2.28 cents per fl oz; *P* < .001), or 28.7%; for taxed family size drinks, it was 1.55 cents per fluid ounce (95% CI, 1.37 to 1.74 cents per fl oz; *P* < .001), a 50.6% increase. There was no statistically significant interaction by neighborhood income level for price changes. In low-income neighborhoods, taxed beverage prices increased by 1.98 cents per fluid ounce (95% CI, 1.47 to 2.49 cents per fl oz; *P* < .001), or 32.8%, while prices in neighborhoods with all other levels of income increased 2.24 cents per fluid ounce (95% CI, 1.75 to 2.74 cents per fl oz; *P* < .001), or 34.9%. There were no significant changes in price per fluid ounce among taxed and nontaxed beverages in neighboring counties compared with Baltimore. (Energy drink price results and 12-month results for changes in calories and grams of sugar not previously reported are provided in eAppendix 2 and eAppendix 4 the Supplement.)

**Table 1.** Changes in Mean Beverage Price per Fluid Ounce at Small Independent Stores 24 Months Post–Tax Implementation

Characteristic	Price, mean (SD), ¢/fl oz				Change in price per fl oz, %^a^	Tax passed through to prices, %^b^	Difference-in-differences, estimate (95% CI)^c^
	Philadelphia (intervention, with tax)		Baltimore (comparison, no tax)				
	Baseline	24 mo	Baseline	24 mo			
**All beverages**							
Taxed^d^	5.95 (2.25)	7.81 (2.43)	6.25 (2.15)	6.13 (2.20)	33.3	137.3	2.06 (1.75 to 2.38)^e^
Not taxed^d^	6.29 (3.04)	6.25 (3.17)	6.56 (2.91)	6.62 (2.79)	−1.7	NA	−0.11 (−0.95 to 0.74)
**By sweetener type**^f^							
Sugar (taxed)	5.92 (2.29)	7.76 (2.44)	6.24 (2.14)	6.11 (2.19)	32.7	135.3	2.03 (1.68 to 2.37)^e^
Artificial (taxed)	6.11 (2.01)	8.14 (2.33)	6.31 (2.20)	6.25 (2.29)	36.1	147.8	2.22 (1.35 to 3.08)^e^
**Individual-sized containers**^g^							
Taxed	6.73 (1.86)	8.67 (1.94)	6.95 (1.75)	6.93 (1.76)	28.7	132.2	1.98 (1.69 to 2.28)^e^
Not taxed	7.80 (3.75)	7.84 (3.71)	8.00 (2.99)	7.97 (2.79)	0.9	NA	0.07 (−1.36 to 1.50)
**Family size containers (>36 oz)**^g^							
Taxed	3.06 (0.59)	4.55 (0.82)	3.31 (0.61)	3.22 (0.60)	50.6	103.7	1.55 (1.37 to 1.74)^e^
Not taxed	5.08 (1.42)	4.64 (1.13)	4.63 (1.19)	4.62 (1.05)	−7.3	NA	−0.37 (−0.92 to 0.18)
**Store location: low-income**^h^							
Taxed	5.71 (2.21)	7.51 (2.43)	6.32 (2.02)	6.14 (2.12)	32.8	131.9	1.98 (1.47 to 2.49)^e^
Not taxed	6.09 (2.58)	6.11 (3.01)	6.43 (2.79)	6.41 (2.72)	0.5	NA	0.03 (−1.22 to 1.28)
**Store location: other-income**^h^							
Taxed	6.25 (2.27)	8.29 (2.35)	6.20 (2.22)	6.12 (2.26)	34.9	149.7	2.24 (1.75 to 2.74)^e^
Not taxed	6.53 (3.49)	6.45 (3.40)	6.65 (3.00)	6.80 (2.84)	−3.4	NA	−0.22 (−1.64 to 1.20)
**All beverages: PA border counties**							
Taxed	5.93 (2.18)^i^	7.12 (29.17)^i^	6.25 (2.15)	6.13 (2.20)	22.0	NA	1.31 (−0.88 to 3.49)
Not taxed	5.59 (2.43)^i^	5.39 (2.58)^i^	6.56 (2.91)	6.62 (2.79)	−4.9	NA	−0.27 (−1.01 to 0.48)

^a^ Percentage change is calculated by dividing the difference-in-differences coefficient by the sum of the intercept and coefficient for Philadelphia. The numerator represents the change in price per fluid ounce 24 months posttax using Baltimore as a control, and the denominator is the mean price per fluid ounce in Philadelphia at baseline.

^b^ Percentage of tax passed through to customer is calculated by dividing the difference-in-differences estimate by 1.5 ¢/fl oz.

^c^ *P *values and 95% CIs were Bonferroni corrected using 2 corrections for main analyses and sweetener type and 4 corrections for beverage size and income.

^d^ Taxed refers to beverages covered under Philadelphia’s 1.5 cent per fluid ounce beverage tax on sugar- and artificially sweetened beverages implemented January 1, 2017. Not taxed refers to beverages not covered under Philadelphia’s beverage tax.

^e^ *P* < .001.

^f^ Sugar-sweetened beverages are any taxed beverages that contain sugar as an ingredient and artificially sweetened beverages are any taxed beverages that contain only artificial sweeteners.

^g^ Beverages are individual-sized if ≤36 fl oz and family-sized if >36 fl oz.

^h^ Income based on census tract–level data from 2014 5-year American Community Survey estimates. Census tracts with 30% or more of the population living in poverty are considered low income and the rest are other income. The Philadelphia and Baltimore analyses include 37 small, independent stores in low-income census tracts and 41 small, independent stores in other-income census tracts.

^i^ Values are for PA border counties, which refers to stores in the 3 Pennsylvania counties neighboring Philadelphia (Bucks, Montgomery, Delaware).

### Changes in Volume of Beverages Purchased

Purchase assessments were made at 63 stores in Baltimore and 58 stores in Philadelphia. The final number of customer purchase assessments at each time point was 2038 at baseline (894 in low-income neighborhoods) and 2700 posttax (1054 in low-income neighborhoods; eAppendix 1 in the Supplement). Of 4738 total customer purchases, 4351 (91.8%) were by customers older than 18 years; 1950 customers (41.2%) identified as women, 3185 customers [67.2%] identified as Black, and 1006 [21.2%] as White (Table 2; eAppendix 3 in the Supplement). Although a small number of stores were not continuously assessed because of closure or diminished customer traffic between baseline and posttax assessment, the proportion of stores located in low-income census tracts remained consistent (eAppendix 3 in the Supplement). There was a 6.12–fl oz decline (95% CI, −9.88 to −2.37 fl oz; *P* < .001), or a 41.9% decrease, in the ounces of taxed beverages purchased per person in Philadelphia compared with Baltimore, and no significant change in the ounces of nontaxed beverages purchased (all volume results appear in Table 3). The reduction in taxed beverage purchases was driven by a 6.17–fl oz decline (95% CI, −9.69 to −2.64 fl oz; *P* < .001) in SSBs, a 47.3% decrease. There were no significant changes in the purchases of artificially sweetened beverages, although few were purchased overall (89 purchases). For taxed individual-sized drinks, the volume purchased per person declined by 3.48 fl oz (95% CI, −6.42 to −0.54; *P* = .003), or 33.7%; the decline in volume purchased per person for taxed family-sized drinks was not significant (−2.31 fl oz; 95% CI, −5.24 to 0.62 fl oz; *P* = .10).

**Table 2.** Customers of Small Independent Stores in Philadelphia and Baltimore

	Philadelphia (intervention, with tax) customers, No. (%)		*P* value^a^	Baltimore (comparison, no tax) customers, No. (%)		*P* value^a^
	Baseline (n = 796)	24 mo (n = 1108)		Baseline (n = 1242)	24 mo (n = 1592)	
Gender						
Men	438 (55.9)	667 (60.9)	.03	718 (57.8)	927 (58.6)	.21
Women	345 (44.0)	429 (39.1)		524 (42.2)	652 (41.2)	
Other	1 (0.1)	0		0	4 (0.3)	
Race^b^						
White	208 (28.1)	306 (28.3)	<.001	207 (16.8)	285 (18.2)	.03
Black	504 (68.2)	639 (59.1)		886 (72.1)	1156 (73.6)	
Other	27 (3.7)	136 (12.6)		136 (11.1)	129 (8.2)	
Hispanic ethnicity	51 (6.6)	98 (9.0)	.05	48 (3.9)	79 (5.0)	.16
Highest level of education						
Less than high school	77 (9.8)	96 (9.0)	<.001	186 (15.1)	176 (11.2)	.001
High school or GED	359 (45.7)	387 (36.4)		529 (42.9)	649 (41.5)	
Some college or Associate’s degree	130 (16.6)	208 (19.6)		246 (20.0)	312 (19.9)	
College degree or higher	219 (27.9)	371 (34.9)		271 (22.0)	428 (27.3)	
Age, y						
13-17	82 (10.4)	91 (8.3)	.11	88 (7.1)	93 (5.9)	.19
≥18	703 (89.6)	1005 (91.7)		1152 (92.9)	1491 (91.4)	
Visited stores in low-income neighborhood	356 (44.7)	463 (41.8)	.20	538 (43.3)	591 (37.1)	.001
City residents	761 (97.1)	1029 (93.0)	<.001	1143 (92.0)	1366 (85.8)	<.001
Shopping frequency^c^						
1 visit/mo	108 (14.1)	151 (13.7)	<.001	222 (18.0)	347 (21.9)	.02
2-3 visits/mo	102 (13.4)	82 (7.4)		153 (12.4)	210 (13.3)	
1-2 visits/wk	248 (32.5)	190 (17.2)		245 (19.9)	294 (18.6)	
3-6 visits/wk	101 (13.2)	209 (19.0)		206 (16.7)	250 (15.8)	
1 visit/d	95 (12.4)	228 (20.7)		196 (15.9)	189 (11.9)	
2-3 visits/d	82 (10.7)	163 (14.8)		131 (10.6)	186 (11.7)	
≥4 visits/d	28 (3.7)	79 (7.2)		78 (6.3)	108 (6.8)	
Who was this purchase for?						
Only you	559 (74.1)	853 (77.6)	<.001	788 (63.5)	1061 (66.7)	.15
Share	190 (25.2)	213 (19.4)		385 (31.0)	441 (27.7)	
Someone else	5 (0.7)	33 (3.0)		67 (5.4)	89 (5.6)	
Total spent on purchase, mean (SD), $	6.07 (7.19)	6.39 (6.00)	.31	7.71 (8.98)	8.34 (9.34)	.07
No. of items purchased, mean (SD)	2.66 (1.99)	2.39 (2.16)	.005	2.63 (2.32)	2.50 (2.22)	.11
Purchased a high-sugar food item or a sweetened beverage	452 (56.8)	529 (47.7)	<.001	622 (50.1)	834 (52.4)	.22
Calories of high-sugar food or sweetened beverages purchased by sugar buyers, Mean (SD)	528 (522)	442 (441)	.01	465 (510)	427 (458)	.13

Abbreviation: GED, General Educational Development.

^a^ Significance for continuous measures is calculated for the within-city difference from baseline using a *t* test. Significance for independent distribution of categories within cities from baseline is calculated with χ^2^ tests. Values may not total 100% due to missing values or rounding.

^b^ Institutional review board permission to waive consent required only allowing 3 racial categories for self-identification (ie, Black, White, and Other).

^c^ The cut points for the store frequency variable (how often do you visit the store) are based on the distribution of the data and differ from the cut points used in our prior paper looking at the association of a sweetened beverage tax with changes in beverage prices and purchases at independent stores 1 year after tax implementation.^14^

**Table 3.** Change in Beverage Volume Purchased per Person at Small Independent Stores 24 Months After the Philadelphia Beverage Tax

Characteristic	Volume sales, mean (SD), fl oz				% Change^a^	Difference-in-differences, estimate (95% CI), fl oz^b^
	Philadelphia (intervention, with tax)		Baltimore (comparison, no tax)			
	Baseline	24 mo	Baseline	24 mo		
**All beverages**						
Taxed^c^	14.31 (22.50)	9.02 (18.41)	10.45 (19.64)	11.53 (23.39)	−41.9	−6.12 (−9.88 to −2.37)^d^
Not taxed^c^	8.69 (23.92)	7.45 (27.34)	4.24 (15.50)	3.95 (17.05)	−5.7	−0.55 (−4.34 to 3.25)
**By sweetener type**^e^						
Sugar (taxed)	13.03 (20.98)	8.48 (18.26)	7.93 (17.25)	10.01 (22.21)	−47.3	−6.17 (−9.69 to −2.64)^d^
Artificial (taxed)	0.45 (4.37)	0.34 (2.48)	0.52 (3.78)	0.60 (6.38)	−42.5	−0.21 (−0.91 to 0.50)
**Individual-sized containers**^f^						
Taxed	10.40 (15.01)	7.56 (14.35)	7.77 (13.15)	9.13 (16.95)	−33.7	−3.48 (−6.42 to −0.54)^g^
Not taxed	4.79 (12.26)	4.46 (9.65)	2.76 (8.07)	2.33 (7.22)	−2.8	−0.16 (−2.07 to 1.75)
**Family sized containers (>36 oz)**^f^						
Taxed	3.91 (18.58)	1.44 (12.09)	2.68 (15.11)	2.33 (16.86)	−56.0	−2.31 (−5.24 to 0.62)
Not taxed	3.90 (20.89)	2.99 (25.52)	1.51 (13.36)	1.63 (15.54)	−21.1	−0.88 (−4.38 to 2.63)
**Store location: low-income**^h^						
Taxed	16.26 (24.98)	11.27 (21.71)	8.29 (17.24)	11.45 (23.67)	−43.3	−7.05 (−12.97 to −1.14)^g^
Not taxed	4.72 (17.39)	6.37 (22.05)	4.99 (13.69)	2.59 (9.95)	24.4	1.89 (−2.74 to 6.52)
**Store location: other-income**^h^						
Taxed	12.73 (20.17)	7.40 (15.43)	12.10 (21.16)	11.57 (23.24)	−40.3	−5.29 (−11.21 to 0.63)
Not taxed	11.90 (27.72)	8.22 (30.58)	3.67 (16.74)	4.76 (20.06)	−31.9	−3.95 (−10.40 to 2.50)
**Customer: lower education**^i^						
Taxed	16.35 (24.11)	10.25 (20.20)	11.46 (20.93)	11.93 (24.04)	−41.4	−6.92 (−12.53 to −1.31)^j^
Not taxed	5.18 (14.88)	7.73 (35.29)	4.14 (16.70)	4.35 (20.27)	28.6	1.67 (−3.85 to 7.18)
**Customer: higher education**^i^						
Taxed	11.81 (20.32)	8.18 (17.29)	9.21 (17.80)	10.76 (20.20)	−34.3	−4.65 (−10.57 to 1.27)
Not taxed	13.25 (31.49)	7.65 (19.72)	4.47 (13.83)	3.62 (12.89)	−14.5	−1.92 (−8.40 to 4.56)

^a^ Percentage change is calculated by dividing the difference-in-differences coefficient by the sum of the intercept and coefficient for Philadelphia. The numerator represents the change in volume purchased 24 months posttax using Baltimore as a control, and the denominator is the mean volume purchased in Philadelphia at baseline.

^b^ Analyses were Bonferroni corrected using 2 corrections for main analyses and sweetener type and 4 corrections for beverage size, income, and education.

^c^ Taxed refers to beverages covered under Philadelphia’s 1.5 cent per fluid ounce beverage tax on sugar- and artificially sweetened beverages implemented January 1, 2017. Not taxed refers to beverages not covered under Philadelphia’s beverage tax.

^d^ *P* < .001.

^e^ Sugar-sweetened beverages are any taxed beverages that contain sugar as an ingredient and artificially sweetened beverages are any taxed beverages that contain only artificial sweeteners.

^f^ Beverages are individual-sized if 36 fl oz or less and family-sized if more than 36 fl oz.

^g^ *P* = .008.

^h^ Income based on census tract–level data from 2014 5-year American Community Survey estimates. Census tracts with 30% or more of the population living in poverty are considered low income and the rest are other income. Of the total 4738 customer purchase assessments, 1948 were collected at small, independent stores located in low-income census tracts and 2790 were collected at stores in other-income census tracts.

^i^ Based on self-report of highest level of education. Low education levels include customers with a high school degree, General Educational Development diploma, or less, while high education levels include customers with some college or more. Of the total 4738 customer purchase assessments, 2459 were collected among customers reporting low education and 2185 were collected among customers reporting high education. Ninety-four customers missing values for education were dropped from education-stratified analyses.

^j^ *P* = .004.

Neither neighborhood income nor customer education significantly moderated volume sales of taxed or nontaxed beverages. These showed that stores in low-income neighborhoods had a significant 43.3% decline in the volume of taxed beverages purchased (−7.05 fl oz; 95% CI, −12.97 to −1.14 fl oz; *P* = .005), while the 40.3% decline in neighborhoods with other income levels was not significant (−5.29 fl oz; 95% CI, −11.21 to 0.63 fl oz; *P* = .11). In exploratory stratified analyses among shoppers with lower education levels, those in Philadelphia purchased 41.4% fewer taxed fluid ounces (−6.92 fl oz; 95% CI, −12.53 to −1.31 fl oz; *P* = .002) compared with Baltimore, while there was no significant difference among those with higher education. The elasticity estimate (not including tax avoidance) using the observed price and volume changes was −1.26 (eAppendix 3 in the Supplement).

### Changes in Calories and Spending on Beverages and High-Sugar Foods

In the baseline sample, customers purchased about 3 items during each shopping trip that included a food or beverage (mean [SD] purchases: Philadelphia, 2.66 [1.99]; Baltimore, 2.63 [2.32]) and spent approximately $7 (mean [SD] purchase amount: Philadelphia, $6.07 [7.19]; Baltimore, $7.72 [8.98]). Of those purchases, 52.7% included either a SSB or a high-sugar food (Philadelphia, 56.8%; Baltimore, 50.1%) (Table 2).

Changes in total calories purchased per person are in the Figure, Table 4, and eAppendix 4 in the Supplement; changes in grams of sugar are in eAppendix 4 in the Supplement. There was a 69-calorie decrease (95% CI, −132 to −5 calories; *P* = .04) in the total calories purchased from SSBs and high-sugar foods combined, a 22.6% decline. The grams of sugar from these items declined by 19.9 g (95% CI, −31.7 to −8.2 g; *P* = .002), or 34.1% per person (eAppendix 4 in the Supplement). Neither neighborhood income nor customer education significantly moderated total calories or grams of sugar sold. In stratified exploratory analyses among low-income neighborhood shoppers, the reduction in total calories purchased per person from SSBs or high-sugar foods was not significant (−86 calories; 95% CI, −191 to 20 calories; *P* = .08; or −25.8%), but the total grams of sugar purchased from SSBs and high-sugar foods declined significantly by 36.3% (−23.7 g; 95% CI, −44.5 to −3.0 g; *P* = .01) (eAppendix 4 in the Supplement). There were no significant changes among shoppers from neighborhoods with other income levels.

**Figure.**
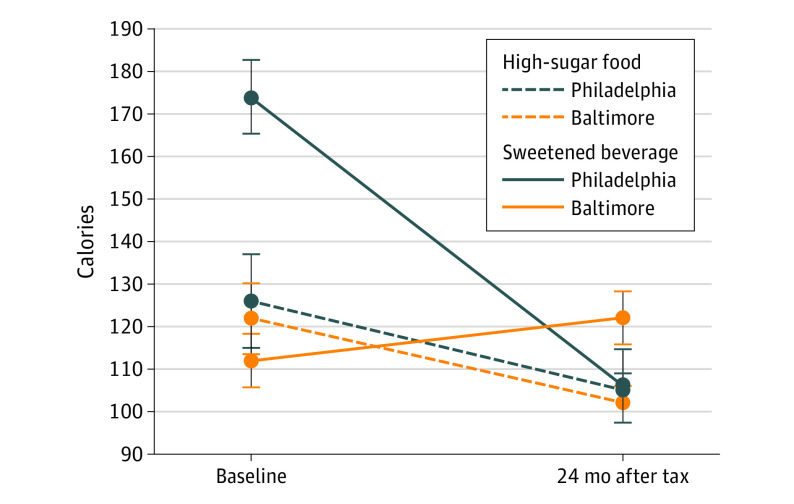
Changes in Calories per Purchase From Beverages and High-Sugar Foods in Philadelphia and Baltimore 24 Months After a Beverage Tax Analyses are based on 4738 customer purchase assessments made at 63 stores in Baltimore and 58 stores in Philadelphia. The number of customer purchase assessments at each time point was 2038 at baseline (894 in low-income neighborhoods) and 2700 posttax (1054 low-income neighborhoods).

**Table 4.** Changes in Total Calories Purchased per Person From High-Sugar Foods and Beverages 24 Months After Philadelphia Beverage Tax

Characteristics	Calories, mean (SD), No.				Change, %^a^	Difference-in-differences, estimate (95% CI), calories^b^
	Philadelphia (intervention, with tax)		Baltimore (comparison, no tax)			
	Baseline	24 mo	Baseline	24 mo		
**All stores**						
High-sugar food	126 (361)	105 (293)	122 (345)	102 (298)	9.9	12 (−36 to 61)
Sweetened beverages	174 (293)	106 (223)	112 (230)	122 (255)	−42.1	−75 (−112 to −37)^c^
High-sugar food or sweetened beverage	300 (473)	211 (376)	233 (430)	224 (394)	−22.6	−69 (−132 to −5)^d^
**Store location: low-income**^e^						
High-sugar food	112 (324)	119 (291)	110 (335)	101 (300)	9.5	12 (−66 to 90)
Sweetened beverages	205 (341)	143 (286)	85 (185)	131 (279)	−46.7	−96 (−161 to −30)^f^
High-sugar food or sweetened beverages	316 (477)	262 (416)	195 (391)	232 (418)	−25.8	−86 (−191 to 20)
**Store location: other-income**^e^						
High-sugar food	138 (388)	95 (294)	130 (353)	102 (297)	10.6	13 (−72 to 98)
Sweetened beverages	148 (244)	80 (157)	132 (257)	116 (239)	−38.2	−59 (−120 to 2)
High-sugar food or sweetened beverages	287 (469)	174 (341)	262 (455)	219 (380)	−17.8	−49 (−159 to 61)
**Customer: low education**^g^						
High-sugar food	103 (314)	135 (354)	130 (330)	103 (315)	52.6	53 (−22 to 128)
Sweetened beverages	207 (325)	127 (258)	121 (237)	133 (288)	−45.0	−94 (−156 to −33)^c^
High-sugar food or sweetened beverages	310 (462)	262 (428)	251 (423)	236 (431)	−14.5	−45 (−145 to 55)
**Customer: high education**^g^						
High-sugar food	157 (414)	76 (225)	109 (368)	101 (280)	−30.4	−47 (−135 to 41)
Sweetened beverages	131 (242)	90 (193)	101 (220)	108 (205)	−27.1	−44 (−106 to 19)
High-sugar food or sweetened beverages	288 (491)	166 (325)	210 (442)	209 (348)	−29.5	−94 (−208 to 22)

^a^ Percentage change is calculated by dividing the difference-in-differences coefficient by the sum of the intercept and coefficient for Philadelphia. The numerator represents the change in calories from high-sugar foods and sugar-sweetened beverages purchased 24 months posttax using Baltimore as a control, and the denominator is the mean calories purchased in Philadelphia at baseline.

^b^ *P *values and confidence intervals were Bonferroni corrected using 2 corrections each for store income and customer education.

^c^ *P* < .001.

^d^ *P* = .03.

^e^ Income based on census-tract-level data from 2014 5-year American Community Survey estimates. Census tracts with 30% or more of the population living in poverty are considered low income and the rest are other income. Of the total 4738 customer purchase assessments, 1948 were collected at small, independent stores located in low-income census tracts and 2790 were collected at stores in other-income census tracts.

^f^ *P* = .001.

^g^ Based on self-report of highest level of education. Low education includes those with a high school degree, General Educational Development diploma, or less, while high education includes those with some college or more. Of the total 4738 customer purchase assessments, 2459 were collected among customers reporting low education and 2185 were collected among customers reporting high education. Ninety-four customers missing values for education were dropped from education-stratified analyses. All models include a random intercept for store location.

Customers with lower education reduced the total grams of sugar purchased per person from SSBs and high-sugar foods by 30.2% (−19.2 g; 95% CI, −38.2 to −0.2 g; *P* = *.*03) (eAppendix 4 in the Supplement), but reductions in calories were not statistically significant (−45 calories; 95% CI, −145 to 55 calories). Customers with higher education had similar reductions in the total grams of sugar (−18.6 g; 95% CI, −38.8 to 1.6 g; *P* = .06; or −32.7%) and calories (−93 calories; 95% CI, −204 to 17 calories; or −29.4%) purchased per person. These results are similar when comparing baseline to 12 months (eAppendix 4 in the Supplement).

There was no significant change in total spending posttax (eAppendix 4 in the Supplement). There was a significant increase in the frequency that customers reported buying SSBs in neighboring counties (0.19; 95% CI, 0.04 to 0.34; *P* = .02) (scale in eAppendix 3 in the Supplement). Using the survey scale midpoints, this translated to less than 1 additional trip outside the city per month compared with baseline.

## Discussion

This study examined the association between the Philadelphia beverage tax in small independent stores 2 years posttax and the change in beverage prices and volume and food purchases. Two years after tax implementation, 137% of the beverage tax was passed through to prices, the volume of taxed beverages purchased had a 41.9% decline, an estimated 6.12 fewer fluid ounces purchased per person, and the number of calories purchased from beverages and high-sugar foods declined by 69 calories purchased per person. Although a number of prior studies in Philadelphia have shown increased prices and purchase declines,^11,14,15,20,21^ this is the first study, to our knowledge, in a US taxed jurisdiction to show sustained, 2-year association of a beverage tax and no evidence of substitution to high-sugar foods among independent store shoppers.

These results extend our prior 1-year analysis, finding higher pass-through (137% vs 120%) and similar declines in taxed beverages (42% vs 39%).^14^ The evaluation of Mexico’s 1-peso-per-liter excise tax on SSBs similarly found larger increases 2 years after implementation.^23^ Although there was no significant moderation by income or education level, our exploratory stratified analyses (like our 1-year analysis^14^) revealed larger absolute declines in taxed beverage purchases among customers shopping in low-income neighborhoods (−7.1 fl oz) and among individuals with lower education levels (−6.9 fl oz) compared with the overall decline (−6.1 fl oz). In addition, absolute declines in calories purchased from beverages and high-sugar foods were larger among customers shopping in low-income neighborhoods (−86 calories vs −69 calories overall). These results, combined with the tax overshifting and no evidence of substitution toward high-sugar foods, suggests the tax may lead to more health gains among people living in lower-income areas or with lower education in the longer term.^34,35^

Unlike our prior research,^14^ this study did not find an increase in total spending. We observed a small self-reported increase in frequency of buying SSBs in neighboring counties across store types. The strengths of this study include use of a real-world, natural experiment design with a nonneighboring control city, a large panel of independent stores and large number of objective customer purchases from a diverse sample, longer-term outcome data, and examination of potential food substitution.

### Limitations

This study has several limitations. First, our data do not include evening and weekend purchases, which may limit generalizability. Second, this study captured purchases at independent stores, and we do not know whether independent store customers switched to purchasing taxed beverages at stores where less of the tax is passed through to prices, such as supermarkets.^15^ Third, we were unable to objectively assess potential cross-border shopping at independent stores and were therefore unable to incorporate that into our elasticity calculation. Fourth, customer purchase results are based on repeated cross-sections and unobservable factors may have changed differentially between sites over time, although sociodemographic characteristics of Philadelphia and Baltimore were similar over time and adjusted models did not affect the overall results. Fifth, we assumed the previously documented parallel trends for large chain retailers in Philadelphia and Baltimore^15^ were similar to the trends in our sample, but we could not test it directly. Sixth, we often relied on storeowner and clerk reports of store prices when prices were not posted, although we do not expect this to differ between cities. Seventh, although our response rate in Baltimore (59%) was comparable with other studies,^36^ our response rate in Philadelphia was lower (23%). Eighth, our data do not allow us to adjust for the volume of customers shopping at each store. Ninth, we had to estimate the nutrition values for 13% of items using the Food and Nutrient Database for Dietary Studies, and our substitution analyses only examined calories from sweetened foods (not all foods). Tenth, a small number of artificially sweetened beverage purchases were made, making it difficult to draw conclusions on the tax’s influence on those drinks. Finally, we were unable to assess the influence of the tax on independent store revenue, although other research suggests sweetened beverage taxes are not associated with job losses.^37,38^

## Conclusions

This cross-sectional study found an association between a tax on sweetened beverages in Philadelphia, Pennsylvania, and a decline in the volume of sweetened beverages purchased in small independent stores. These results suggest that beverage excise taxes can lead to longer-term, sustained reductions in purchases of sweetened drinks and calories from sugar in small independent stores, with large reductions in lower-income areas and among populations with lower education levels.

## Notes

Supplement. **eAppendix 1.** Study Methods **eAppendix 2.** Beverage Price Results **eAppendix 3.** Volume Purchase Results **eAppendix 4.** High Sugar Food and Spending Analyses
