# Supplementary material for: Association of a Sweetened Beverage Tax With Purchases of Beverages and High-Sugar Foods at Independent Stores in Philadelphia
Source: JAMA Netw Open. 2021 Jun 15;4(6):e2113527. doi: 10.1001/jamanetworkopen.2021.13527 (PMC8207239; doi:10.1001/jamanetworkopen.2021.13527)
Supplement: Supplement. — eAppendix 1. Study Methods eAppendix 2. Beverage Price Results eAppendix 3. Volume Purchase Results eAppendix 4. High Sugar Food and Spending Analyses [file jamanetwopen-e2113527-s001.pdf]

Supplemental Online Content

Bleich SN, Dunn CG, Soto MJ, et al. Association of a sweetened beverage tax with purchases of beverages and high-sugar foods at independent stores in Philadelphia. *JAMA Netw Open.* 2021;4(6):e2113527. doi:10.1001/jamanetworkopen.2021.13527

eAppendix 1. Study Methods

eAppendix 2. Beverage Price Results

eAppendix 3. Volume Purchase Results

eAppendix 4. High Sugar Food and Spending Analyses

This supplemental material has been provided by the authors to give readers additional information about their work.

© 2021 Bleich SN et al. *JAMA Network Open.*

eAppendix 1. Study Methods

eAppendix 1: Study Methods

1.1. Beverage classification

Philadelphia beverage tax criteria: Beverages subject to Philadelphia's excise tax include soda, fruit drinks (not including 100% juice), sports drinks, flavored waters, energy drinks, pre-sweetened coffee or tea, and non-alcoholic beverages intended to be mixed into an alcoholic drink. Beverages that are not subject to Philadelphia's excise tax include: unsweetened drinks including those to which a purchaser can add sugar or request the addition of sugar (e.g., black coffee purchased at an independent store), baby formulas, beverages that meet the definition of medical food, any product for which more than 50% of its volume is milk or fresh fruit, vegetable, or a combination, and any syrup or other concentrate that a customer combines with other ingredients to create a beverage (e.g., powdered drink mixes like Kool-Aid).

1.2. Nutrient and serving size coding

We conducted online searches to identify the nutrient content and serving size for each the items in the high-sugar food categories: candy (n=265 at baseline, n=563 post-tax), sweet snacks (n=343 baseline, 830 post-tax) and pure sugar (n=7 baseline, n=11 post-tax). We searched for specific brand and flavor combinations identified by data collectors (e.g., Brand: Tropical Fantasy, Flavor: Guava) and prioritized nutrition information provided on brand websites (e.g., pepsicobeveragefacts.com). If brand websites did not provide nutrition information, we searched online retailers (e.g., Walmart.com, Amazon.com), and recorded nutrition information only if the retail listing included a photograph of the nutrition label. Next, we searched the USDA FoodData Central database (fdc.nal.usda.gov) for brand and flavor-specific nutrition information. If nutrition data for the brand/flavor combination were not available from any of the above resources, we used the USDA FoodData Central database to find the closes approximation of the food or beverage based on the level of detail provided by data collectors. For example, we were unable to find nutrition information for Lady Linda Honey Buns from our first three sources and instead selected a generic "honey bun" from the FoodData Central database. We were able to identify the nutrient content of foods and beverages for 3,112 (52%) items from brand websites, 1,624 (27%) items from online retailers, 488 (8%) items from the USDA FoodData Central, and 764 (13%) items closely approximated from the USDA FoodData Central.

For beverages, we recorded serving size in fluid ounces; if fluid ounces were not available, we recorded serving size in milliliters. For foods, we recorded serving size in grams. For all items, we recorded the kilocalories and grams of total sugar per serving to create a per-unit value. Using the number of servings per item (recorded by data collectors during purchase assessments), we were able to calculate the total calories and total grams of sugar per item.

1.3. Beverage price methods: Store recruitment

Sample size and exclusions. We collected data from 161 independent stores at baseline (53 in Philadelphia, 60 in Baltimore, 48 in neighboring counties). During the study, 36 stores closed or refused to continue participating and were replaced with similar stores near the original ones. Additionally, ten stores changed ownership, but remained in the same physical location. After excluding those stores that changed ownership or did not continuously provide data, the final analytic sample sizes for complete case analysis were 35 stores in Philadelphia (21 low-income, 14 other-income), 43 stores in Baltimore (16 low-income, 27 other-income), and 38 stores in PA counties neighboring Philadelphia (16 low-income, 22 other-income) bringing the total number of stores to 116 (see **Figure 1.3.a).** Census data were used to examine the comparability of the neighborhoods where price data were collected in Philadelphia and Baltimore (**Table 1.3.b**). Data showed neighborhood sociodemographic characteristics were similar.

Among all independent stores at baseline and 24 months, we collected data from 103 brand-size combinations including 66 (64.08%) sugar-sweetened beverages, 12 (11.65%) artificially-sweetened beverages, and 25 (24.27%) unsweetened beverages. Energy drinks (n=278 prices from 5 brand-size combinations collected at 65 stores) were excluded from price analyses due to their higher mean price per fluid ounce (energy drinks mean = 23.64 cents per fluid ounce across baseline and post-tax) compared to other taxed beverages (means ranged from 6.08 - 6.25 cents per fluid ounce). (See **eAppendix**

© 2021 Bleich SN et al. *JAMA Network Open.*

Table 2.1. for changes in energy drink prices at independent stores in Philadelphia compared to Baltimore at 24-month follow up).

Figure 1.3.a. Store recruitment and study flow for price data

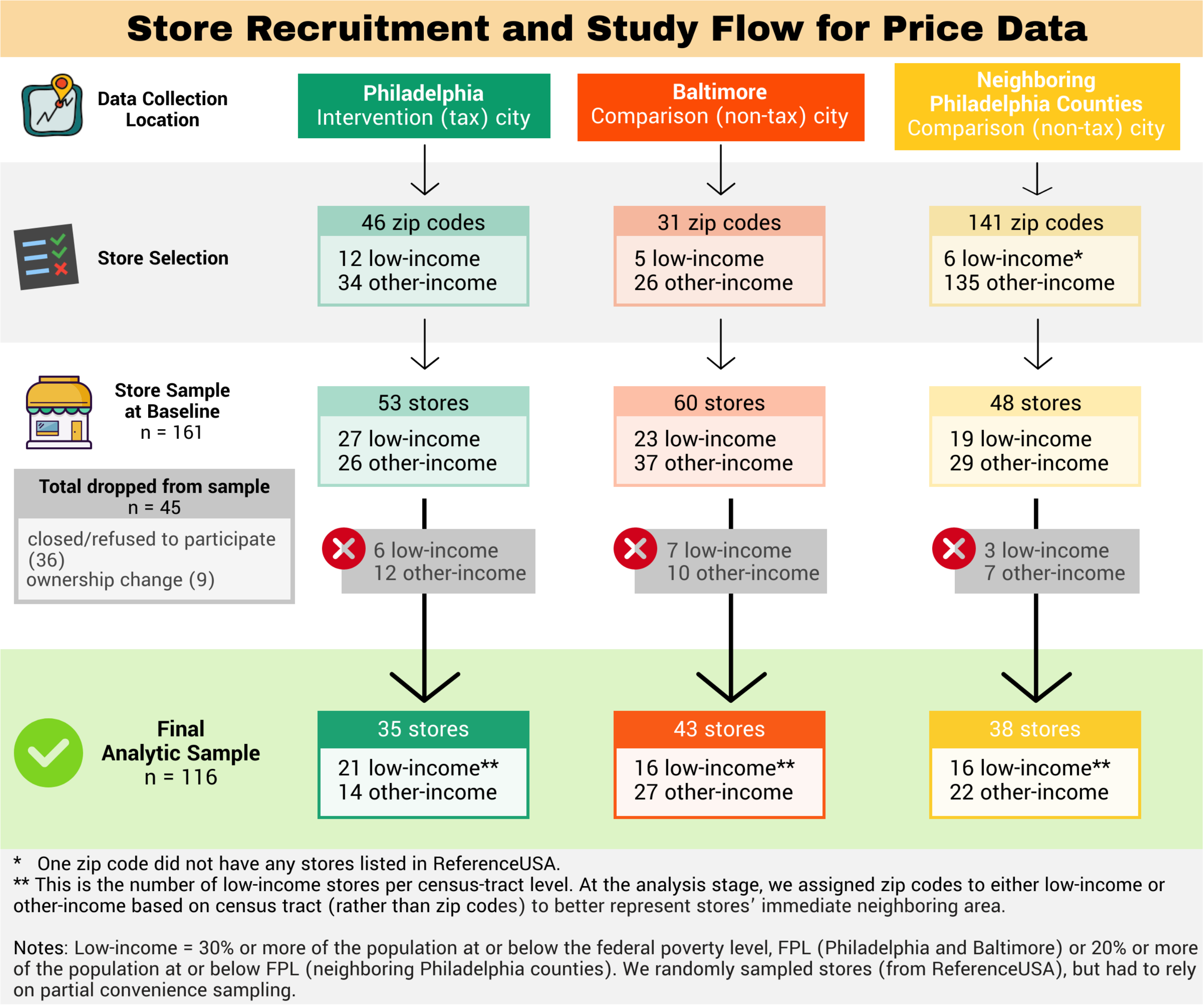

© 2021 Bleich SN et al. *JAMA Network Open.*

Table 1.3.b Neighborhoods where price data were collected

	Philadelphia		Baltimore	
	Low-Income	*Other-Income*	*Low-Income*	*Other-Income*
**# Tracts**	20	18	12	21
**% Age**				
<20	26.2	16.2	30.2	18.3
20-34	34.1	36.6	25.8	36.4
35-64	29.3	35.4	34.8	35.9
65+	10.3	11.8	9.3	9.5
**% Race**				
Black	61.5	25.0	72.3	50.4
White	22.5	54.3	16.0	37.9
Hispanic	4.9	9.9	3.3	4.9
Other	11.2	10.7	8.4	6.8
**% Education**				
Less than HS	18.6	11.3	22.0	12.8
HS, GED, some college	58.4	44.0	55.7	44.5
University degree+	22.9	44.7	22.4	42.7
**% Federal poverty level**				
Mean	41.2	17.3	39.0	18.0
Std. Dev.	11.4	7.2	8.0	7.7
**Households**				
Total households	1,385.2	1,820.1	943.8	1,347.6
Average household size	2.5	2.2	2.7	2.3
Percent households with 1+ child	22.6	17.9	31.2	21.3
Total family households	589.5	743.7	475.2	575.7
Average family size	3.5	3.1	3.6	3.1
**% Occupied housing units with 1+ vehicles**	45.1	67.8	52.4	73.0

Notes: Tract level estimates were averaged for each income and city group based on 2016 5-year American Community Survey data. A household consists of all people who occupy a housing unit, including for example, roommates living together, a person living alone, or a family. A family household consists of a group of two or more people who are related residing together.

1.4. Additional details about customer purchase assessments

At baseline we captured 1,265 taxed and 584 non-taxed beverage purchases at independent stores in Philadelphia and Baltimore. Beverages were coded as either sugar-sweetened (59.8%, including drinks containing a mix of sugar and artificial sweetener), artificially-sweetened (3.8%), or unsweetened (34.2%). For our analyses of taxed beverages, we included 159 (2.2%) beverages that were sweetened, but we were unable to determine their sweetener type. This included taxed beverages (such as 7-Eleven Big Gulp beverages where the exact beverage flavor was unspecified) and non-taxed beverages (such as cappuccinos, which are at least 50% milk, and therefore excluded from the tax). These beverages are excluded from analyses of sweetener type, since we are unable to determine if these are sugar- or artificially-sweetened.

© 2021 Bleich SN et al. *JAMA Network Open.*

Figure 1.4. Participant recruitment for volume data

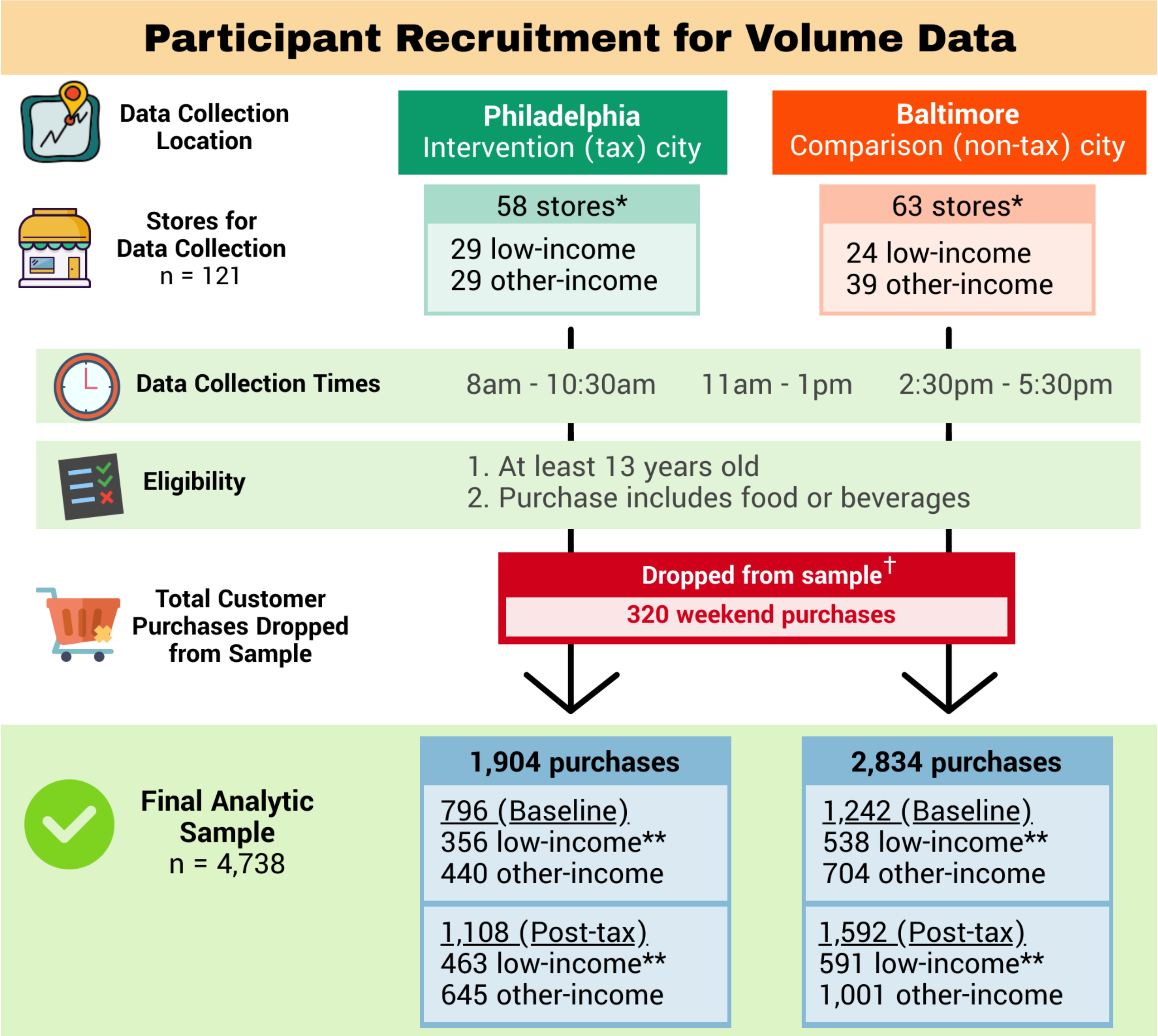

* Stores were sampled at the zip code level.
** This is the number of low-income stores per census-tract level. At the analysis stage, we assigned zip codes to either low-income or other-income based on census tract (rather than zip codes) to better represent stores' immediate neighboring area.
† Prior to analysis, we excluded 320 weekend purchases (6.3% of 5,058 collected at independent stores), as Baltimore staffing shortages only enabled the collection of 35 weekend purchases.

Notes: Low-income = 30% or more of the population at or below the federal poverty level (FPL) in Philadelphia and Baltimore.

© 2021 Bleich SN et al. *JAMA Network Open.*

eAppendix 2: Beverage Price Results

2.1. Changes in energy drink prices at independent stores 24 months after a Philadelphia beverage tax

Energy drinks were excluded from the main analyses because they had a much higher price per fluid ounce than other beverages. We found no change in the mean price per fluid ounce or the overall price among these beverages.

Table 2.1. Changes in energy drink prices at independent stores 24 months after a Philadelphia beverage tax

Characteristic	Price per fl oz mean (SD), ¢/fl oz				Change in ¢/fl oz, %[b]	Tax passed through to prices, %[c]	Difference-in-differences, estimate (95% CI)	Adjusted *p*-value[d]
	Philadelphia (intervention, with tax)		Baltimore (comparison, no tax)					
	**Baseline**	**24 months**	**Baseline**	**24 months**				
Taxed[a]	21.88 (7.20)	25.08 (10.55)	23.89 (6.91)	23.50 (6.90)	16.6	241.9	3.63 (-0.65 to 7.91)	0.114
	**Price mean (SD), $**				**Change in price, %[b]**	**Tax passed through to prices, %[c]**	**Difference-in-differences, estimate (95% CI)**	**Adjusted *p*-value[d]**
	**Philadelphia (intervention, with tax)**		**Baltimore (comparison, no tax)**					
	**Baseline**	**24 months**	**Baseline**	**24 months**				
Taxed[a]	2.52 (0.88)	2.93 (0.76)	2.75 (0.70)	2.77 (0.78)	15.6	N/A	0.38 (-0.01 to 0.78)	0.056

SOURCE: Analysis of price data from 5 beverage-size combinations in 65 small, independent stores, 31 in Philadelphia and 34 in Baltimore, before and two years after implementation of the tax.

NOTES: [a]"Taxed" refers to beverages covered under Philadelphia's 1.5 cent per fluid ounce beverage tax on sugar- and artificially-sweetened beverages implemented Jan 1, 2017. [b]Percent change is calculated by dividing the difference-in-differences coefficient by the sum of the intercept and coefficient for Philadelphia. The numerator represents the change in price or price per fluid ounce 24 months post-tax using Baltimore as a control, and the denominator is the mean price or price per fluid ounce in Philadelphia at baseline. [c]Percent of taxed passed through to customer is calculated by dividing the difference-in-differences estimate by 1.5¢/fl oz. [d]*P* values and confidence intervals were Bonferroni corrected using 2 corrections.

© 2021 Bleich SN et al. *JAMA Network Open.*

eAppendix 3. Volume Purchase Results

3.1. Model selection and sensitivity analyses

We identified covariates that we hypothesized would influence the standard error of our model including: gender, race, ethnicity, education, age, who the purchase was for, frequency visiting the store, city residency, and total amount spent. The table below presents results adjusted for these covariates along with the unadjusted model that we present in the main paper and a complete case model, which excludes observations missing any of the covariates in our adjusted model (n=622, 13.1%). Our plan was to conduct unadjusted analyses because of the difference-in-differences design. We did, however, identify covariates a priori that were likely associated with our outcome to see if there were gains in efficiency. The adjustments did not meaningfully change the parameter estimates, standard errors, or our conclusions, so we report the unadjusted models in the main paper.

Table 3.1 Model selection and sensitivity analyses

	n	**% Change**	**Difference-in-differences, estimate (95% CI), fl oz**	**Standard Error**	***p*-value**
**Crude**	4738	-41.9	-6.12 (-9.29, -2.96)	1.61	<.001
**Crude Complete Case**	4116	-44.1	-6.76 (-10.22 to -3.30)	1.77	<.001
**Adjusted model**	4116	-50.8	-7.48 (-10.97 to –3.99)	1.78	<.001

© 2021 Bleich SN et al. *JAMA Network Open.*

3.2. Customers of independent stores in Philadelphia and Baltimore at baseline and 6, 12 and 24 months

At baseline, customers were mostly male, Black, over age 18, and had a high school degree/GED or higher. Customer purchase assessments were not collected in neighboring PA counties due to funding constraints and a short pre-tax data collection window. Our original target sample size based on power calculations was 3,260 participants across Philadelphia and Baltimore across pre/post tax time points.

Table 3.2. Customers of independent stores in Philadelphia and Baltimore at baseline and 6, 12 and 24 months

	**Customers, N (%)**													
	**Philadelphia (intervention, with tax)**							**Baltimore (comparison, no tax)**						
	**Baseline (n=796)**	**6 months (n=760)**	***P***	**12 months (n=827)**	***P***	**24 months (n=1108)**	***P***	**Baseline (n=1242)**	**6 months (n=1392)**	***P***	**12 months (n=1766)**	***P***	**24 months (n=1592)**	***P***
**Gender**				*		*								
Men	438 (55.9)	410 (55.1)		513 (62.6)		667 (60.9)		718 (57.8)	822 (59.1)		1009 (57.3)		927 (58.6)	
Women	345 (44.0)	334 (44.9)	.856	305 (37.2)	.010	429 (39.1)	.027	524 (42.2)	569 (40.9)	.577	751 (42.6)	.477	652 (41.2)	.211
Other	1 (0.1)	0 (0.0)		1 (0.1)		0 (0.0)		0 (0.0)	1 (0.1)		2 (0.1)		4 (0.3)	
**Race**				***		***			***		**		*	
White	208 (28.1)	189 (25.7)		132 (16.2)		306 (28.3)		207 (16.8)	204 (14.7)		316 (18.0)		285 (18.2)	
Black	504 (68.2)	508 (69.0)	.212	627 (76.7)	<.001	639 (59.1)	<.001	886 (72.1)	1109 (80.0)	<.001	1304 (74.5)	.003	1156 (73.6)	.033
Other	27 (3.7)	39 (5.3)		58 (7.1)		136 (12.6)		136 (11.1)	73 (5.3)		131 (7.5)		129 (8.2)	
**Hispanic ethnicity**	51 (6.6)	32 (4.4)	.070	54 (6.7)	>.999	98 (9.0)	.052	48 (3.9)	47 (3.4)	.479	76 (4.3)	.577	79 (5.0)	.162
**Highest level of education**		**		**		***			**		*		**	
Less than high school	77 (9.8)	97 (13.0)		101 (12.5)		96 (9.0)		186 (15.1)	155 (11.2)		225 (13.1)		176 (11.2)	
High school or GED	359 (45.7)	290 (38.9)		388 (47.8)		387 (36.4)		529 (42.9)	633 (45.6)		709 (41.1)		649 (41.5)	
Some college or Associate's degree	130 (16.6)	161 (21.6)	.005	156 (19.2)	.004	208 (19.6)	<.001	246 (20.0)	245 (17.7)	.003	321 (18.6)	.011	312 (19.9)	.001
College degree or higher	219 (27.9)	198 (26.5)		166 (20.5)		371 (34.9)		271 (22.0)	354 (25.5)		469 (27.2)		428 (27.3)	
**Age, y**		**		***										

© 2021 Bleich SN et al. *JAMA Network Open.*

	Customers, N (%)													
	Philadelphia (intervention, with tax)							Baltimore (comparison, no tax)						
	Baseline (n=796)	6 months (n=760)	*P*	12 months (n=827)	*P*	24 months (n=1108)	*P*	Baseline (n=1242)	6 months (n=1392)	*P*	12 months (n=1766)	*P*	24 months (n=1592)	*P*
13-17	82 (10.4)	46 (6.2)	.003	43 (5.3)	<.001	91 (8.3)	.113	88 (7.1)	92 (6.6)	.643	95 (5.4)	.054	93 (5.9)	.187
≥18	703 (89.6)	697 (93.8)		776 (94.7)		1005 (91.7)		1152 (92.9)	1300 (93.4)		1668 (94.6)		1491 (94.1)	
**Visited stores in low-income neighborhood**				***									**	
	356 (44.7)	349 (45.9)	.647	456 (55.1)	<.001	463 (41.8)	.202	538 (43.3)	643 (46.2)	.139	767 (43.4)	.970	591 (37.1)	.001
**City residents**		**		*		***					***		***	
	761 (97.1)	714 (94.2)	.006	782 (94.6)	.012	1029 (93.0)	<.001	1143 (92.0)	1279 (91.9)	.890	1541 (87.3)	<.001	1366 (85.8)	<.001
**Shopping frequency**		***		***		***			***		***		*	
1 visit/mo	108 (14.1)	114 (15.4)	<.001	98 (11.9)	<.001	151 (13.7)	<.001	222 (18.0)	237 (17.2)	<.001	399 (22.7)	<.001	347 (21.9)	.016
2-3 visits/mo	102 (13.4)	80 (10.8)		47 (5.7)		82 (7.4)		153 (12.4)	128 (9.3)		206 (11.7)		210 (13.3)	
1-2 visits/wk	248 (32.5)	134 (18.1)		125 (15.2)		190 (17.2)		245 (19.9)	249 (18.0)		346 (19.7)		294 (18.6)	
3-6 visits/wk	101 (13.2)	121 (16.3)		162 (19.6)		209 (19.0)		206 (16.7)	203 (14.7)		268 (15.2)		250 (15.8)	
1 visit/d	95 (12.4)	117 (15.8)		151 (18.3)		228 (20.7)		196 (15.9)	165 (12.0)		169 (9.6)		189 (11.9)	
2-3 visits/d	82 (10.7)	126 (17.0)		138 (16.7)		163 (14.8)		131 (10.6)	219 (15.9)		227 (12.9)		186 (11.7)	
≥4 visits/d	28 (3.7)	50 (6.7)		104 (12.6)		79 (7.2)		78 (6.3)	178 (12.9)		146 (8.3)		108 (6.8)	
**Who was this purchase for?**		***		***		***			***		***			
Only you	559 (74.1)	526 (69.8)	<.001	573 (69.7)	<.001	853 (77.6)	<.001	788 (63.5)	1015 (73.0)	<.001	1249 (70.8)	<.001	1061 (66.7)	.154
Share	190 (25.2)	194 (25.7)		214 (26.0)		213 (19.4)		385 (31.0)	340 (24.5)		425 (24.1)		441 (27.7)	
Someone else	5 (0.6)	34 (4.5)		35 (4.3)		33 (3.0)		67 (5.4)	35 (2.5)		91 (5.2)		89 (5.6)	
**Total spent on purchase, mean (SD), $**									***					
	6.07 (7.19)	6.20 (8.16)	.731	5.68 (7.05)	.269	6.39 (6.00)	.308	7.71 (8.98)	6.31 (8.09)	<.001	7.47 (8.36)	.456	8.34 (9.34)	.074

© 2021 Bleich SN et al. *JAMA Network Open.*

	Customers, N (%)												
	Philadelphia (intervention, with tax)							Baltimore (comparison, no tax)					
	Baseline (n=796)	6 months (n=760)	*P*	12 months (n=827)	*P*	24 months (n=1108)	*P*	Baseline (n=1242)	6 months (n=1392)	*P*	12 months (n=1766)	*P*	24 months (n=1592)	*P*
**No. of items purchased, mean (SD)**						**			*		***			
	2.66 (1.99)	2.47 (2.38)	.078	2.47 (2.27)	.070	2.39 (2.16)	.005	2.63 (2.32)	2.40 (2.56)	.013	2.31 (1.84)	<.001	2.50 (2.22)	.114
**Purchased a high-sugar food item**														
	189 (23.7)	160 (21.1)	.203	187 (22.6)	.589	233 (21.0)	.159	290 (23.3)	342 (24.6)	.464	361 (20.4)	.059	328 (20.6)	.079
**Purchased a sweetened beverage**		**		***		***			***		***		**	
	361 (45.4)	279 (36.7)	.001	282 (34.1)	<.001	375 (33.8)	<.001	428 (34.5)	590 (42.4)	<.001	732 (41.4)	<.001	627 (39.4)	.007
**Purchased a high-sugar food item or a sweetened beverage**				**		***			***		*			
	452 (56.8)	396 (52.1)	.064	405 (49.0)	.002	529 (47.7)	<.001	622 (50.1)	823 (59.1)	<.001	965 (54.6)	.014	834 (52.4)	.223
**Sugar of high-sugar food purchased by sugar buyers, mean (SD), g[a]**														
	24.6 (64.8)	N/A	N/A	20.3 (34.6)	.212	21.6 (44.3)	.406	26.1 (55.4)	N/A	N/A	20.7 (106.8)	.191	20.7 (49.5)	.055
**Sugar of sweetened beverages purchased by sugar buyers, mean (SD), g[a]**				**		***					*			
	78.3 (87.3)	N/A	N/A	62.3 (75.9)	.004	55.9 (72.8)	<.001	56.5 (72.3)	N/A	N/A	65.6 (104.2)	.038	59.4 (80.8)	.467
**Sugar of high-sugar food or sweetened beverages purchased by sugar buyers, mean (SD), g[a]**				***		***								
	102.9 (101.4)	N/A	N/A	82.5 (77.7)	<.001	77.5 (70.7)	<.001	82.6 (85.5)	N/A	N/A	86.4 (145.4)	.507	80.1 (91.7)	.600
**Calories of high-sugar food purchased by**											***		*	
	223 (457)	N/A	N/A	201 (349)	.434	220 (393)	.917	243 (457)	N/A	N/A	170 (362)	<.001	194 (389)	.033

© 2021 Bleich SN et al. *JAMA Network Open.*

	Customers, N (%)													
	Philadelphia (intervention, with tax)							Baltimore (comparison, no tax)						
	Baseline (n=796)	6 months (n=760)	*P*	12 months (n=827)	*P*	24 months (n=1108)	*P*	Baseline (n=1242)	6 months (n=1392)	*P*	12 months (n=1766)	*P*	24 months (n=1592)	*P*
**sugar buyers, mean (SD)[a]**														
**Calories of sweetened beverages purchased by sugar buyers, mean (SD)[a]**				*		***								
	306 (332)	N/A	N/A	251 (299)	.012	222 (279)	<.001	223 (284)	N/A	N/A	246 (329)	.132	233 (313)	.533
**Calories of high-sugar food or sweetened beverages purchased by sugar buyers, mean (SD)[a]**				*		**					*			
	528 (522)	N/A	N/A	452 (411)	.018	442 (441)	.006	465 (510)	N/A	N/A	416 (451)	.048	427 (458)	.134

SOURCE: Analysis of customer purchase assessments (N=9,483) from 130 small, independent stores.

NOTES: Significance for continuous measures is calculated for the within-city standardized mean difference from baseline using a t-test. Significance for independent distribution of categories within cities from baseline is calculated with Chi-Square Test of Independence. *$p < .05$, **$p < .01$, ***$p < .001$, Values may not sum to 100% due to missing values or rounding. The cut points for the store frequency variable (how often do you visit the store) are based on the distribution of the data and differ from the cut points used in our prior paper looking at the association of a sweetened beverage tax with changes in beverage prices and purchases at independent stores one year after tax implementation. [14]

[a] Sugar buyers refers to someone who purchased a sweetened beverage or a high-sugar food.

© 2021 Bleich SN et al. *JAMA Network Open.*

3.3. Stores in the customer purchase assessment sample by city and wave

We conducted customer purchase assessments at a total of 58 independent stores in Philadelphia and 63 independent stores in Baltimore. The stores where customer purchase assessments were conducted were largely similar over time, though there were some differences due to store closures or differences in store traffic. The proportion of stores within categories of store type or income remained consistent.

Table 3.3. Stores in the customer purchase assessment sample by city and wave

	Philadelphia (intervention, with tax)			Baltimore (comparison, no tax)		
	**Total**	**Baseline**	**24 months**	**Total**	**Baseline**	**24 months**
**Independent Stores**	58	33	40	63	48	42
**Store location: Income level, *N* (%)**[a]						
Low	31 (50.8%)	19 (55.9%)	19 (47.5%)	25 (38.5%)	18 (37.5%)	15 (35.7%)
Other	30 (49.2%)	15 (44.1%)	21 (52.5%)	40 (61.5%)	30 (62.5%)	27 (64.3%)
**Purchase Assessments per Store**						
Mean (SD)	30.5 (26.6)	16.9 (19.7)	27.3 (31.9)	47.7 (45.0)	23.6 (30.1)	37.9 (27.9)
Median (IQR)	23 (8 – 42)	8 (4 – 28)	17.5 (7 – 28)	35 (14 – 71)	12 (7– 30.5)	36 (16 – 49)

SOURCE: Census-tract-level data from 2014 5-year American Community Survey estimates.
NOTES: SD=Standard deviation. IQR=Inter-quartile range.
[a]Census tracts with 30% or more of the population living in poverty are considered "low-income" and the rest are "other-income". The cross-section of stores at each timepoint is not completely overlapping so column totals do not sum to the overall total.

© 2021 Bleich SN et al. *JAMA Network Open.*

3.4. Price elasticity of demand for taxed beverages

Elasticity was calculated based on the following equation: Elasticity=% change in volume/%change in price. The change in price was observed from our price audits (which do not include every beverage a store sells) and the change in volume was based on the customer purchase assessment data from purchases within Philadelphia and Baltimore.

These elasticity estimates do not account for possible tax avoidance behavior in PA counties neighboring Philadelphia, which would likely reduce the volume change estimates. Customer purchase assessments were not collected in neighboring PA counties due to funding constraints and a short pre-tax data collection window.

Table 3.4. Price elasticity of demand for taxed beverages 24 months after a Philadelphia beverage tax

	Price Change (¢/fl oz)	Change in ¢/fl oz, %	Store Audits (n)	Change in Volume Sales, %	Purchase Assessments (n)	Elasticity
**Overall Sample**	2.06	33.3	78	-41.9	4,738	-1.26

© 2021 Bleich SN et al. *JAMA Network Open.*

eAppendix 4. High-sugar food and spending analyses

4.1. Change in calories purchased from high-sugar foods and beverages 12 months after a Philadelphia beverage tax

Table 4.1 Changes in calories purchased from high-sugar foods and beverages 12 months after a Philadelphia beverage tax

Overall, there was no significant change in the total calories of high-sugar foods purchased in Philadelphia compared to Baltimore post tax. There was a significant decrease in total calories purchased from sweetened beverages, and from high-sugar foods and sweetened beverages combined. The difference-in-differences estimates for calories purchased were not moderated by neighborhood income or customer education.

	Calories purchased, mean (SD)					
	Philadelphia (intervention, with tax)		Baltimore (comparison, no tax)		Adj. % Change[c]	Difference-in-differences, estimate (95% CI)[d]
	Baseline	12 months	Baseline	12 months		
**All stores**						
High-sugar food	126 (361)	98 (264)	122 (345)	93 (281)	2.9	3 (-42, 49)
Sweetened beverages	174 (293)	123 (244)	112 (230)	135 (272)	-34.6	-63**(-102, -24)
High-sugar food or sweetened beverage	300 (473)	221 (366)	233 (430)	227 (393)	-21.1	-64*(-125, -2)
**Store location: low-income[a]**						
High-sugar food	112 (324)	94 (232)	110 (335)	102 (296)	-2.2	-3 (-78, 73)
Sweetened beverages	205 (341)	140 (260)	85 (185)	120 (256)	-35.2	-72*(-137, -8)
High-sugar food or sweetened beverages	316 (477)	234 (360)	195 (391)	222 (387)	-24.5	-80 (-183, 23)
**Store location: other-income[a]**						
High-sugar food	138 (388)	104 (300)	130 (353)	86 (269)	7.2	9 (-66, 85)
Sweetened beverages	148 (244)	102 (221)	132 (257)	145 (284)	-36.6	-56 (-119, 8)
High-sugar food or sweetened beverages	287 (469)	206 (373)	262 (455)	231 (397)	-17.9	-49 (-151, 53)
**Customer: lower education[b]**						
High-sugar food	103 (314)	109 (285)	130 (330)	96 (275)	36.4	37 (-31, 105)
Sweetened beverages	207 (325)	140 (258)	121 (237)	141 (258)	-38.0	-79**(-140, -18)
High-sugar food or sweetened beverages	310 (462)	249 (386)	251 (423)	237 (374)	-14.9	-46 (-141, 49)
**Customer: higher education[b]**						
High-sugar food	157 (414)	83 (231)	109 (368)	85 (276)	-19.3	-29 (-111, 52)
Sweetened beverages	131 (242)	98 (223)	101 (220)	127 (288)	-27.3	-44 (-110, 21)
High-sugar food or sweetened beverages	288 (491)	181 (334)	210 (442)	212 (406)	-24.0	-74 (-183, 34)

SOURCE: Analysis of Philadelphia and Baltimore included 4,631 customer purchase assessments at 119 small, independent stores.

NOTES: Baseline data are from 2016.

[a]Income based on census-tract-level data from 2014 5-year American Community Survey estimates. Census tracts with 30% or more of the population living in poverty are considered “low-income” and the rest are “other-income”. Of the total 4,631 customer purchase assessments, 2,117 were collected at small, independent stores located in low-income census tracts and 2,514 were collected at stores in other-income census tracts.

[b]Based on self-report of highest level of education. “Lower education” includes those with a high school degree, GED, or less, while “higher education” includes those with some college or more. Of the total 4,631 customer purchase assessments, 2,574 were collected among customers reporting “lower education” and 1,988 were collected among customers reporting “higher education”. 79 customers missing values for education were dropped from education-stratified analyses. All models include a random intercept for store location.

© 2021 Bleich SN et al. *JAMA Network Open.*

[c]Percent change is calculated by dividing the difference-in-differences coefficient by the sum of the intercept and coefficient for Philadelphia. The numerator represents the change in calories from high-sugar foods and SSBs 12 months post-tax using Baltimore as a control, and the denominator is the mean calories purchased in Philadelphia at baseline.
[d]*p* values and confidence intervals were Bonferroni corrected using 2 corrections each for store location neighborhood income and customer education. *$p<0.05$, **$p<0.01$, ***$p<0.001$

© 2021 Bleich SN et al. *JAMA Network Open.*

4.2. Change in sugar purchased from high-sugar foods and beverages 12 and 24 months after a Philadelphia beverage tax

Table 4.2.a Change in sugar purchased from high-sugar foods and beverages 12 months after a Philadelphia beverage tax

Overall, there was no significant change in the total grams of sugar of high-sugar foods purchased in Philadelphia compared to Baltimore post tax. There was a significant decrease in total grams of sugar from sweetened beverages and high-sugar foods and sweetened beverages combined. The difference-in-differences estimates for grams of sugar purchased were not moderated by neighborhood income or customer education.

	Sugar purchased, mean (SD), g					
	Philadelphia (intervention, with tax)		Baltimore (comparison, no tax)		Adj. % Change[c]	Difference-in-differences, estimate (95% CI)[d]
	Baseline	12 months	Baseline	12 months		
**All stores**						
High-sugar food	14.0 (50.3)	9.9 (26.2)	13.1 (41.3)	11.3 (79.6)	-10.4	-1.4 (-9.7, 6.9)
Sweetened beverages	44.5 (76.3)	30.5 (61.6)	28.3 (58.4)	35.9 (83.7)	-39.7	-18.3**(-29.0, -7.5)
High-sugar food or sweetened beverages	58.4 (91.8)	40.4 (68.3)	41.3 (73.3)	47.2 (115.5)	-33.8	-19.8*(-33.7, -6.0)
**Store location: low-income[a]**						
High-sugar food	11.3 (42.1)	9.8 (24.3)	11.6 (39.8)	10.7 (35.0)	-1.0	-0.1 (-9.1, 8.8)
Sweetened beverages	52.7 (89.6)	34.9 (66.1)	21.1 (46.5)	32.5 (84.9)	-42.3	-22.1**(-40.2, -3.9)
High-sugar food or sweetened beverages	64.0 (100.3)	44.7 (72.9)	32.7 (62.0)	43.2 (92.6)	-34.3	-22.2*(-43.1, -1.3)
**Store location: other-income[a]**						
High-sugar food	16.2 (56.0)	10.1 (28.5)	14.2 (42.5)	11.8 (101.3)	-18.6	-2.7 (-19.4, 14.0)
Sweetened beverages	37.8 (63.0)	25.1 (55.2)	33.8 (65.6)	38.5 (82.7)	-40.4	-15.7 (-33.1, 1.6)
High-sugar food or sweetened beverages	53.9 (84.3)	35.2 (61.8)	47.9 (80.2)	50.3 (130.3)	-34.5	-17.9 (-42.4, 6.6)
**Customer: lower education[b]**						
High-sugar food	10.1 (38.3)	10.4 (25.5)	14.2 (39.8)	10.0 (29.8)	36.2	3.6 (-4.1, 11.3)
Sweetened beverages	53.0 (84.9)	34.7 (65.5)	30.9 (60.6)	37.3 (76.4)	-40.6	-21.3**(-38.2, -4.4)
High-sugar food or sweetened beverages	63.1 (95.0)	45.1 (71.5)	45.1 (73.6)	47.3 (81.0)	-29.3	-18.4 (-37.2, 0.5)
**Customer: higher education[b]**						
High-sugar food	19.0 (62.3)	9.2 (27.8)	11.3 (43.5)	12.6 (114.1)	-43.2	-7.3 (-27.8, 13.2)
Sweetened beverages	33.7 (63.4)	24.3 (55.6)	25.2 (55.6)	34.2 (91.8)	-35.5	-14.5 (-33.5, 4.4)
High-sugar food or sweetened beverages	52.7 (88.6)	33.5 (63.5)	36.6 (73.2)	46.9 (147.2)	-37.7	-21.4 (-49.7, 6.8)

SOURCE: Authors' analysis of Philadelphia and Baltimore included 4,631 customer purchase assessments at 119 small, independent stores.
NOTES: Baseline data are from 2016.
[a]Income based on census-tract-level data from 2014 5-year American Community Survey estimates. Census tracts with 30% or more of the population living in poverty are considered "low-income" and the rest are "other-income". Of the total 4,631 customer purchase assessments, 2,117 were collected at small, independent stores located in low-income census tracts and 2,514 were collected at stores in other-income census tracts.
[b]Based on self-report of highest level of education. "Lower education" includes those with a high school degree, GED, or less, while "higher education" includes those with some college or more. Of the total 4.631 customer purchase assessments, 2,574 were collected among customers reporting "lower education" and 1,978 were collected among customers reporting "higher education". 79 customers missing values for education were dropped from education-stratified analyses. All models include a random intercept for store location.
[c]Percent change is calculated by dividing the difference-in-differences coefficient by the sum of the intercept and coefficient for Philadelphia. The numerator represents the change in grams of sugar from high-sugar food and SSBs purchased 12 months post-tax using Baltimore as a control, and the denominator is the mean grams of sugar purchased in Philadelphia at baseline.

© 2021 Bleich SN et al. *JAMA Network Open.*

[d] p values and confidence intervals were Bonferroni corrected using 2 corrections each for store location neighborhood income and customer education. *p<0.05, **p<0.01, ***p<0.001

© 2021 Bleich SN et al. *JAMA Network Open.*

Table 4.2.b Change in sugar purchased from high-sugar foods and beverages 24 months after a Philadelphia beverage tax

Overall, there was no significant change in the total grams of sugar of high-sugar foods purchased in Philadelphia compared to Baltimore post tax. There was a significant decrease in total grams of sugar from sweetened beverages and high-sugar foods and sweetened beverages combined. Education level moderated grams of sugar purchased from high-sugar food (interaction estimate=+12.9; 95% CI 2.8 to 23.0, *p*=.01) such that customers with lower education levels increased their grams of sugar purchased from high-sugar foods more than those with higher education levels, but it did not moderate the grams of sugar purchased from sweetened beverages or from high-sugar food and sweetened beverages combined. The difference-in-differences estimates for grams of sugar purchased was not moderated by neighborhood income.

	Sugar purchased, mean (SD), g					
	Philadelphia (intervention, with tax)		Baltimore (comparison, no tax)		Adj. % Change[c]	Difference-in-differences, estimate (95% CI)[d]
	Baseline	24 months	Baseline	24 months		
**All stores**						
High-sugar food	14.0 (50.3)	10.3 (32.5)	13.1 (41.3)	10.8 (37.3)	4.5	0.6 (-5.4, 6.5)
Sweetened beverages	44.5 (76.3)	26.7 (57.5)	28.3 (58.4)	31.1 (65.6)	-43.8	-19.8***(-29.5, -10.2)
High-sugar food or sweetened beverages	58.4 (91.8)	37.0 (67.2)	41.3 (73.3)	42.0 (77.5)	-34.1	-19.9**(-31.7, -8.2)
**Store location: low-income[a]**						
High-sugar food	11.3 (42.1)	13.7 (41.2)	11.6 (39.8)	11.2 (39.2)	7.0	0.9 (-9.2, 11.1)
Sweetened beverages	52.7 (89.6)	36.7 (75.1)	21.1 (46.5)	33.5 (72.3)	-47.4	-24.9**(-42.0, -7.7)
High-sugar food or sweetened beverages	64.0 (100.3)	50.4 (86.6)	32.7 (62.0)	44.7 (88.0)	-36.3	-23.7*(-44.5, -3.0)
**Store location: other-income[a]**						
High-sugar food	16.2 (56.0)	7.9 (24.1)	14.2 (42.5)	10.6 (36.1)	-4.5	-0.6 (-10.6, 9.4)
Sweetened beverages	37.8 (63.0)	19.5 (39.0)	33.8 (65.6)	29.7 (61.3)	-41.2	-16.2*(-31.9, -0.6)
High-sugar food or sweetened beverages	53.9 (84.3)	27.4 (46.6)	47.9 (80.2)	40.3 (70.6)	-32.0	-16.7 (-35.8, 2.3)
**Customer: lower education[b]**						
High-sugar food	10.1 (38.3)	14.4 (43.1)	14.2 (39.8)	11.5 (42.4)	54.7	5.5 (-4.0, 15.0)
Sweetened beverages	53.0 (84.9)	32.4 (67.5)	30.9 (60.6)	34.2 (74.8)	-45.6	-24.3***(-40.2, -8.4)
High-sugar food or sweetened beverages	63.1 (95.0)	46.8 (79.1)	45.1 (73.6)	45.7 (86.8)	-30.2	-19.2*(-38.2, -0.2)
**Customer: higher education[b]**						
High-sugar food	19.0 (62.3)	6.6 (19.8)	11.3 (43.5)	10.2 (31.2)	-40.8	-6.8 (-17.1, 3.5)
Sweetened beverages	33.7 (63.4)	22.4 (49.0)	25.2 (55.6)	27.3 (52.2)	-31.3	-13.0* (-29.0, -3.0)
High-sugar food or sweetened beverages	52.7 (88.6)	28.9 (56.3)	36.6 (73.2)	37.5 (65.0)	-32.7	-18.6(-38.8, 1.6)

SOURCE: Authors' analysis of Philadelphia and Baltimore included 4,738 customer purchase assessments at 121 small, independent stores.

NOTES: Baseline data are from 2016. All models include a random intercept for store location.

[a]Income based on census-tract-level data from 2014 5-year American Community Survey estimates. Census tracts with 30% or more of the population living in poverty are considered "low-income" and the rest are "other-income". Of the total 4,738 customer purchase assessments, 1,948 were collected at small, independent stores located in low-income census tracts and 2,790 were collected at stores in other-income census tracts.

[b]Based on self-report of highest level of education. "Lower education" includes those with a high school degree, GED, or less, while "higher education" includes those with some college or more. Of the total 4,738 customer purchase assessments, 2,459 were collected among customers reporting "lower education" and 2,185 were collected among customers reporting "higher education". 94 customers missing values for education were dropped from education-stratified analyses.

[c]Percent change is calculated by dividing the difference-in-differences coefficient by the sum of the intercept and coefficient for Philadelphia. The numerator represents the change in grams of sugar from high-sugar food and SSBs purchased 24 months post-tax using Baltimore as a control, and the denominator is the mean grams of sugar purchased in Philadelphia at baseline.

© 2021 Bleich SN et al. *JAMA Network Open.*

[d]*p* values and confidence intervals were Bonferroni corrected using 2 corrections each for store location neighborhood income and customer education. *p<0.05, **p<0.01, ***p<0.001

© 2021 Bleich SN et al. *JAMA Network Open.*

4.3. Change in total spent per customer purchase assessment 24 months after a Philadelphia beverage tax

Overall, there was no significant change in the total amount spent per intercept for all items purchased in Philadelphia compared to Baltimore post tax. The difference-in-differences estimate for total reported spending was not moderated by neighborhood income or customer education.

Table 4.3. Change in total spent[a] per customer purchase assessment 24 months after a Philadelphia beverage tax

	Total spent, mean (SD), $					
	Philadelphia (intervention, with tax)		Baltimore (comparison, no tax)			Difference-in-differences, estimate (95% CI)[e]
	Baseline	24 months	Baseline	24 months	Adj. % Change[d]	
**All stores**	6.07 (7.19)	6.39 (6.00)	7.72 (8.98)	8.34 (9.34)	-1.2	-0.07 (-1.26, 1.14)
**Store location: Low-income[b]**	4.28 (4.22)	5.36 (6.14)	6.22 (6.07)	5.91 (5.47)	12.2	0.54 (-0.64, 1.73)
**Store location: Other-income[b]**	7.53 (8.64)	7.13 (5.78)	8.87 (10.55)	9.77 (10.74)	-13.3	-0.92 (-3.02, 1.18)
**Customer: lower education[c]**	4.25 (3.81)	5.59 (6.51)	6.33 (7.97)	6.91 (7.84)	17.7	0.85 (-0.52, 2.21)
**Customer: higher education[c]**	8.33 (9.42)	7.01 (5.49)	9.63 (9.89)	9.88 (10.51)	-24.6	-1.70 (-3.74,0.33)

SOURCE: Analysis of Philadelphia and Baltimore included 4,738 customer purchase assessments at 121 small, independent stores.
NOTES: Baseline data are from 2016.
[a]Total spending as reported by the customer.
[b]Income based on census-tract-level data from 2014 5-year American Community Survey estimates. Census tracts with 30% or more of the population living in poverty are considered “low-income” and the rest are “other-income”. Of the total 4,738 customer purchase assessments, 1,948 were collected at small, independent stores located in low-income census tracts and 2,790 were collected at stores in other-income census tracts.
[c]Based on self-report of highest level of education. “Lower education” includes those with a high school degree, GED, or less, while “higher education” includes those with some college or more. Of the total 4,738 customer purchase assessments, 2,459 were collected among customers reporting “lower education” and 2,185 were collected among customers reporting “higher education”. 94 customers missing values for education were dropped from education-stratified analyses.
[d]Percent change is calculated by dividing the difference-in-differences coefficient by the sum of the intercept and coefficient for Philadelphia. The numerator represents the change in total spent per customer 24 months post-tax using Baltimore as a control, and the denominator is the mean total spent in Philadelphia at baseline.
[e]*p* values and confidence intervals were Bonferroni corrected using 2 corrections each for store location neighborhood income and customer education.
*p<0.05, **p<0.01, ***p<0.001

© 2021 Bleich SN et al. *JAMA Network Open.*
